# Wernicke’s Encephalopathy: A Precipitation of Glycogen Storage Disease

**DOI:** 10.7759/cureus.31471

**Published:** 2022-11-14

**Authors:** Samah Idris, Aya Abdelgadir Ahmed, Rehab Ali, Hamasat Alsharari, Eman N Ahmed, Farah Deeba

**Affiliations:** 1 Neurological Surgery, Alfaisal University College of Medicine, Riyadh, SAU; 2 Medicine and Surgery, Al Jouf University, Sakaka, SAU; 3 Internal Medicine, Alfaisal University College of Medicine, Riyadh, SAU

**Keywords:** computed tomography, magnetic resonance imaging, cerebellum, alcohol, thiamine deficiency

## Abstract

A 16-year-old Saudi female who is a known case of glycogen storage disease type 1A (GSD1A), presented to the emergency department at King Faisal Specialist Hospital, Riyadh, Saudi Arabia on 15th January 2021 due to a complaint of persistent vomiting. Two weeks after admission, she began developing double vision and progressive leg weakness with intact sensation. She received the primary management to maintain good hydration and was admitted to the ICU for further workup. Over her hospital course, multiple investigations were conducted, the most significant of which was the MRI after sudden ocular deterioration. The result depicted findings classic for Wernicke’s encephalopathy (WE) on MRI. The patient was then started on Thiamine supplementation and MRI performed three weeks later showed significant interval improvement of the parenchymal signal abnormality with complete resolution features of Wernicke's encephalopathy. This complex case emphasizes the need for early recognition and immediate treatment with IV thiamine in such a potential condition that can lead to permanent neurological deficits that present in a non-typical fashion.

## Introduction

Thiamine (Vitamin B1) is a water-soluble vitamin that gets absorbed in the small intestine, i.e., jejunum and ileum. It has a biological half-life of approximately 10-20 days and because the human body has limited tissue storage of thiamine, it must be in continuous supply by food intake. The recommended daily allowance for thiamine in adults should be at least 1.2 mg/day [1]. Thiamine is a cofactor for key enzymes involved in brain energy production such as the Krebs cycle. Brain structures such as the periaqueductal nuclei, mammillary bodies and thalami with high metabolic requirements are especially vulnerable to thiamine deficiency [2]. Since thiamine stores are limited to 20 days, there is a possibility of thiamine deficit which can be categorized as primary and secondary. A primary deficit results from poor diet and a secondary deficit results from cases of increased demand such as hyperthyroidism, pregnancy, stress, fever, or due to defective uptake such as in recurrent vomiting and diarrhea or even defective metabolism such as in liver disorders [3]. While the most common cause of thiamine deficiency is persistent vomiting in alcoholics, it should also be considered in patients with hyperemesis gravidarum, drug-induced hyperlactemia, and acute gastrointestinal illness in already malnourished individuals mainly because it could have a catastrophic onset and less likely to show classical signs on presentation [2]. Thiamine deficiency induces cardiovascular manifestations such as high output heart failure (wet beriberi), gastrointestinal disturbances such as gastrointestinal beriberi, and neurological manifestations the most life-threatening being Wernicke's encephalopathy (WE) [4]. These patients present with weight loss, anorexia, and mental changes such as apathy, confusion, irritability, amnesia, muscle weakness, and cardiomyopathy [3]. All these conditions carry a high rate of morbidity and mortality when unrecognized.

Wernicke's encephalopathy is clinically characterized by the classical triad of ocular abnormalities (horizontal nystagmus, 6th nerve palsy, ptosis and less frequently retinopathy and papilledema), ataxia, and altered level of consciousness [4]. The deficiency of thiamine manifesting as Wernicke's encephalopathy with concurrent optic neuropathy is rare [5]. It has been reported as a presentation of WE in a few anecdotal case reports. The symptoms of ophthalmoplegia (Abducens Palsy) and nystagmus are related to the involvement of the pontine tegmentum, while the change in mental status is related to the involvement of the thalamic or mammillary bodies. Pathological studies have confirmed that neural and vascular damage may occur in the mammillary bodies, periaqueductal areas, and medial nucleus of the thalamus and cause the characteristic brain lesions of WE [6]. Among all neurological investigations, magnetic resonance imaging (MRI) is the gold standard to make a diagnosis of Wernicke’s encephalopathy, which displays as areas of symmetrical increase T2/FLAIR signal. WE can result in permanent brain damage and result in fatality if left untreated. Patients with a high suspicion of WE should be immediately administered IV thiamine, it is the most effective treatment.

## Case presentation

A 16-year-old Saudi female who is a known case of glycogen storage disease type 1A was diagnosed at six months of age. She presented to the ED at King Faisal Specialist Hospital & Research Center on 15th January 2021 complaining of persistent vomiting and elevated lactate level. At the emergency, the patient developed tachycardia with high anion gap metabolic acidosis and electrolyte disturbances such as hypokalemia and hypomagnesemia, secondary to her condition, GSD type 1A. On physical examination, the patient appeared in distress and vitally unstable. Her recorded findings were tachycardia (145 bpm), a high peripheral pulse rate (150 bpm), tachypnea (30 breaths/min), blood pressure of 130/87 mmHg, temperature of 38.5 degrees Celsius, and oxygen saturation of 98% (Table 1). Systemic exams, i.e. cardiovascular and respiratory examinations, were unremarkable. On laboratory investigation, venous blood gas showed high anion gap metabolic acidosis (pH 7.25, paCO2 37, HCO3 21, anion gap 20, lactate level 18) and laboratory findings for electrolytes were positive for hypokalemia (3.3), hypomagnesemia (0.72). The patient was suspected for septicemia and a sepsis workup was ordered. The chest X-ray (CXR) was negative for the source of infection however urinalysis and culture were positive which confirmed the source of infection, therefore, she was started on Tazocin (Piperacillin/tazobactam). Further renal derangement was noted with elevated levels of creatinine (350 micromol/L) and blood urea nitrogen (BUN) (26.3 mg/dL). Nephrology was consulted for the management of acute kidney injury and she was shifted to the ICU and started on a normal saline and bicarbonate infusion. Nephrology deferred the need for renal replacement therapy as creatinine and BUN levels started improving within two days after initiation of normal saline.

**Table 1:** Vital signs and laboratory investigation results for the patient

Investigations	Results	Units	Reference range
Heart rate	145	bpm	60-100
Peripheral pulse rate	150	bpm	60-100
Respiratory rate	30	breath/min	12-20
Blood pressure	130/87	mmHg	120/80
Temperature	36.5	Celsius	36.1-37.2
Oxygen saturation	100	%	80-100
Peripheral blood sugar	69	Mg/dl	72-108
Potassium	3.3	mmol/L	3.6-5.2
Magnesium	0.72	Mg/dl	1.7-2.2
pH	7.26	-	7.35-7.45
Lactate	18	mmol/L	2-4

**Figure 1:**
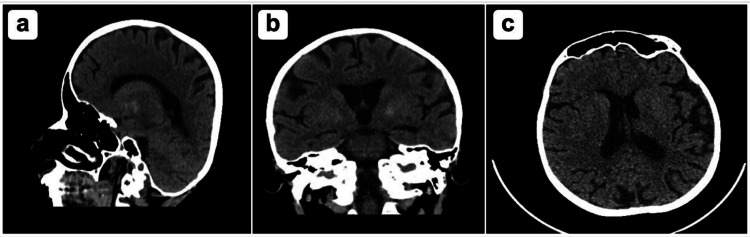
CT brain without contrast showed: A) no acute intracranial hemorrhage or ischemic insult. No gross intracranial masses, mass effect or midline shift. B) Gray and white matter differentiation are preserved. No evolving hydrocephalus. Mild asymmetric hyperdensity in the basal ganglia related to mineralization. C) Hyperpneumatization of left petrous apex. Paranasal sinuses are clear. Partial opacification of the right mastoid air cells. No CT evidence of acute intracranial insult.

She complained of progressive bilateral lower limb weakness without a sensory loss that started four months ago rendering her limited to a walking aid for ambulation. The patient had multiple complaints of difficulty swallowing, dizziness, gait imbalance, and panic attack and was being followed up simultaneously by a multidisciplinary team (MDT) covered by Speech, Language and Pathology, Ophthalmology, Neurology, Mental Health, Physiotherapy and the primary Medical Genetics team. Within two weeks of admission, on 28th January the patient developed new-onset binocular diplopia and an ophthalmology exam demonstrated esotropia, bilateral limitation in abduction, mild limitations in adduction, intact convergence, and horizontal jerk nystagmus at horizontal gaze. This presentation brought in the suspicion for bilateral sixth nerve palsy. A neurological exam for this patient showed normal sensory exam and chronic lower leg weakness with absent reflexes bilaterally and downgoing plantar response. An electromyogram/nerve conduction study (EMG/NCS) was done and it reported complete resolution of previously recorded denervation as well as recording of motor unit potentials (MUP) in muscles that had complete absence of MUPs previously. However, the MUPs recorded still had myogenic morphology which suggested an underlying myopathic process. A computerized tomography brain without contrast was ordered to rule out diplopia and increased intracranial pressure but the result was negative for acute brain insult (Figure 1).

**Figure 2:**
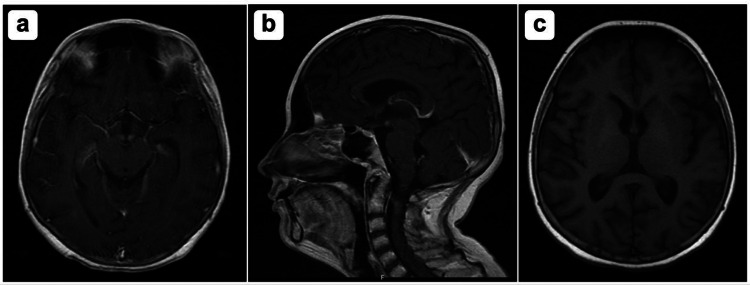
MRI with and without contrast showing: A) bilateral mild swelling and T2 hyperintense edema of the mammillary bodies. Additional symmetrical T2 hyperintense signal is noted along the dorsal medulla, extending to the Ponto medullary junction. B) There is also T2-flair hyperintense signal in the periaqueductal region and mild bilateral periventricular T2 hyperintensity. There is a small area of T2 hyperintense signal abnormality in the cerebellar vermis. C) Corresponding enhancement is noted, mainly in the bilateral mammillary bodies and mild enhancement along the bilateral dorsal medulla. Findings are highly suggestive of Wernicke encephalopathy/thiamine deficiency.

On 30th January, the patient had a sudden marked limitation in abduction and adduction of both eyes, bilateral ophthalmoplegia. A magnetic resonance imaging with and without contrast was done to investigate further. The results showed findings that were highly pathognomonic with Wernicke’s encephalopathy/thiamine deficiency as detailed in Figure 2, as well as showing mild brain volume loss and bilateral mineralization of globus pallidi.

**Figure 3:**
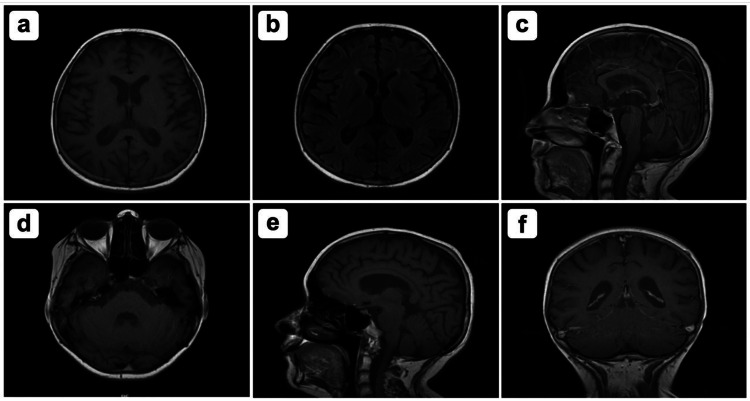
MRI with and without contrast in seven sections indicating: A) interval improvement of the T2 hyperintensity and resolution of the associated enhancement involving bilateral mammillary bodies and dorsal medulla. B) Interval resolution of the abnormal T2-flair hyperintensity involving periaqueductal region. C) Stable mild bilateral periventricular T2 hyperintensity. D) No new focal parenchymal signal abnormality. E) Stable mild diffuse parenchymal volume loss with enlargement of the extra-axial CSF spaces and ventricular system. No evolving hydrocephalus. F) Stable susceptibility artifact of the bilateral Globus Pallidi corresponding to the mineralization on prior brain CT. CSF: Cerebrospinal fluid

The patient was started on 100 mg thiamine supplementation administered via nasogastric tube. MRI with and without contrast was repeated three weeks later which showed significant interval improvement of the parenchymal signal abnormality with complete resolution of the abnormal enhancement related to features of Wernicke's encephalopathy. This patient was kept on thiamine supplementation for the course of three months (Figure 3).

In summary, the patient developed sudden ocular abnormalities, MRI done showed the classic thiamine deficiency findings. This patient demonstrated a complex case of subacute nutritional deficiency due to self-dietary over-restriction on top of an underlying glycogen storage disease. This resulted in severe vitamin deficiencies, at the top of which was thiamine deficiency resulting in Wernicke encephalopathy.

## Discussion

Our patient developed binocular diplopia after two weeks of admission due to thiamine insufficiency in a nonalcoholic condition. Although Wernicke’s encephalopathy is easily preventable, it is often undiagnosed in nonalcoholic patients due to the absence of the classic triad. Numerous situations rather than alcohol have been reported for thiamine deficiency including malnutrition, gastric bypass surgery, parenteral nutrition, gastrointestinal cancer, chemotherapy, hyperemesis gravidarum, thyrotoxicosis, drug-induced hyperlactatemia, and infectious diseases [2,7,8]. In our patient, the symptoms first began with intractable vomiting caused by her primary disorder GSD1A. Furthermore, this repetitive vomiting led to a decrease in body stores of thiamine which was missed until she developed ocular manifestations of nutritional thiamine deficiency. The presentation was exaggerated due to the difficulty feeding and agitated nature of the patient by multiple ongoing factors such as panic attacks, gait instability, and an understandably irritable patient, all of which facilitated nutritional deficit.

In the present patient, MRI with and without contrast showed bilateral mild swelling, T2 hyperintense edema of the mammillary bodies, symmetrical T2 hyperintense signal along the dorsal medulla extending to the Ponto medullary junction and T2 hyperintense signal in the periaqueductal region and mild bilateral periventricular T2 hyperintensity as well as a small area of T2 hyperintense signal abnormality in the cerebellar vermis. Findings are highly suggestive of Wernicke encephalopathy/thiamine deficiency. A previous study that analyzed MRI findings for 56 patients with Wernicke encephalopathy showed typical findings of the disease in alcoholics that were contrast enhancement in the mammillary bodies and thalamus, whereas atypical MR imaging findings in non-alcoholic patients [9]. Furthermore, numerous studies have reported atypical MRI findings in nonalcoholic WE, consisting of T2 and FLAIR signal hyperintensities located in the cerebellum, cranial nerves nuclei, red nuclei, splenium, caudate, putamen, and/or cerebral cortex [8,10-12]. Wernicke’s encephalopathy is a medical emergency that should be suspected in all cases of persistent vomiting and malnutrition with a sudden deficit of VI cranial nerve function or lack of coordination of the ocular movements with or without disturbed consciousness.

## Conclusions

Wernicke's encephalopathy (WE) is a medical emergency. Although WE is commonly viewed in the context of alcoholism, it can be caused by thiamine deficiency secondary to persistent vomiting. This case serves to highlight the fact that the classical triad is not always typical in a clinical setting. All suspected high-risk individuals presenting to the emergency room should receive 200 mg parenteral thiamine before administration of glucose.

## References

[1] Institute of Medicine (1998). Dietary Reference Intakes for Thiamin, Riboflavin, Niacin, Vitamin B6, Folate, Vitamin B12, Pantothenic Acid, Biotin, and Choline.

[2] Antel K, Singh N, Chisholm B, Heckmann JM (2015). Encephalopathy after persistent vomiting: three cases of non-alcohol-related Wernicke's encephalopathy. S Afr Med J.

[3] Porfido D, Guerriero S, Giancipoli G, Vetrugno M, Lefons V, Dicuonzo F (2010). Bilateral sixth nerve palsy as a manifestation of Wernicke’s encephalopathy in a patient with refractory vomiting. Eye Brain.

[4] Donnino M (2004). Gastrointestinal beriberi: a previously unrecognized syndrome. Ann Intern Med.

[5] Yeh WY, Lian LM, Chang A, Cheng CK (2013). Thiamine-deficient optic neuropathy associated with Wernicke's encephalopathy in patients with chronic diarrhea. J Formos Med Assoc.

[6] Halavaara J, Brander A, Lyytinen J, Setälä K, Kallela M (2003). Wernicke's encephalopathy: is diffusion-weighted MRI useful?. Neuroradiology.

[7] Osiezagha K, Ali S, Freeman C (2013). Thiamine deficiency and delirium. Innov Clin Neurosci.

[8] Abbas SA, Abboud H, Chalah MA, Sabbagh C, Ayache SS (2018). Isolated mammillary bodies damage-An atypical presentation of Wernicke syndrome. Behav Sci (Basel).

[9] Manzo G, De Gennaro A, Cozzolino A, Serino A, Fenza G, Manto A (2014). MR imaging findings in alcoholic and nonalcoholic acute Wernicke's encephalopathy: a review. Biomed Res Int.

[10] Bae SJ, Lee HK, Lee JH, Choi CG, Suh DC (2001). Wernicke's encephalopathy: atypical manifestation at MR imaging. Am J Neuroradiol.

[11] Zuccoli G, Motti L (2008). Atypical Wernicke's encephalopathy showing lesions in the cranial nerve nuclei and cerebellum. J Neuroimaging.

[12] Santos Andrade C, Tavares Lucato L, da Graça Morais Martin M, Joaquina Marques-Dias M, Antonio Pezzi Portela L, Scarabôtolo Gattás G, da Costa Leite C (2010). Non-alcoholic Wernicke's encephalopathy: broadening the clinicoradiological spectrum. Br J Radiol.

